# Severe radiation-induced lymphopenia during concurrent chemoradiotherapy for stage III non-small cell lung cancer: external validation of two prediction models

**DOI:** 10.3389/fonc.2023.1278723

**Published:** 2023-11-09

**Authors:** Peter S. N. van Rossum, Celia Juan-Cruz, Barbara Stam, Maddalena M. G. Rossi, Steven H. Lin, Azadeh Abravan, José S. A. Belderbos, Jan-Jakob Sonke

**Affiliations:** ^1^ Department of Radiation Oncology, Netherlands Cancer Institute-Antoni van Leeuwenhoek Hospital, Amsterdam, Netherlands; ^2^ Department of Radiation Oncology, Amsterdam University Medical Centers (UMC), Amsterdam, Netherlands; ^3^ Department of Radiation Oncology, The University of Texas MD Anderson Cancer Center, Houston, TX, United States; ^4^ Division of Cancer Sciences, School of Medical Sciences, Faculty of Biology, Medicine and Health, The University of Manchester, Manchester, United Kingdom; ^5^ Department of Radiotherapy Related Research, The Christie National Health Service (NHS) Foundation Trust, Manchester, United Kingdom

**Keywords:** lung cancer, radiotherapy, chemoradiotherapy, lymphopenia, hematologic toxicity

## Abstract

**Background:**

Severe radiation-induced lymphopenia (RIL) in patients undergoing chemoradiotherapy (CRT) for non-small cell lung cancer (NSCLC) is associated with decreased immunotherapy efficacy and survival. At The Christie and MD Anderson Cancer Center (MDACC), prediction models for lymphopenia were developed in lung and esophageal cancer patients, respectively. The aim of this study was to externally validate both models in patients with stage III NSCLC.

**Methods:**

Patients who underwent concurrent CRT for stage III NSCLC in 2019–2021 were studied. Outcomes were grade ≥3 and grade 4 lymphopenia during CRT. The Christie model predictors for grade ≥3 lymphopenia included age, baseline lymphocyte count, radiotherapy duration, chemotherapy, mean heart and lung doses, and thoracic vertebrae V20Gy. MDACC predictors for grade 4 lymphopenia were age, baseline lymphocyte count, planning target volume (PTV), and BMI. The external performance of both models was assessed.

**Results:**

Among 100 patients, 78 patients (78%) developed grade ≥3 lymphopenia, with grade 4 lymphopenia in 17 (17%). For predicting grade ≥3 lymphopenia, the Christie and MDACC models yielded *c*-statistics of 0.77 and 0.79, respectively. For predicting grade 4 lymphopenia, *c*-statistics were 0.69 and 0.80, respectively. Calibration for the Christie and MDACC models demonstrated moderate and good agreement, respectively.

**Conclusion:**

The PTV-based MDACC prediction model for severe RIL demonstrated superior external performance in NSCLC patients compared to the dosimetry-based Christie model. As such, the MDACC model can aid in identifying patients at high risk for severe lymphopenia. However, to optimize radiotherapy planning, further improvement and external validation of dosimetry-based models is desired.

## Introduction

Non-small cell lung cancer (NSCLC) accounts for approximately 85% of all lung cancer cases and presents as locally advanced (stage III) disease in approximately one-fifth of patients (1, 2). Since the early 1990s, the standard treatment for patients with unresectable stage III NSCLC has consisted of radiotherapy in combination with concurrent platinum-based chemotherapy (3, 4). After a few years, consolidative immunotherapy (i.e., durvalumab) after concurrent chemoradiotherapy (CRT) became the new standard of care in this setting as the PACIFIC trial demonstrated a sustained survival benefit (5, 6). By blocking PD-L1, durvalumab allows the patient’s vital T-lymphocytes to recognize and kill tumor cells.

As these important anti-tumor lymphocytes are the most radiosensitive cells of the hematopoietic system, many get killed during radiotherapy, which puts patients at risk of radiation-induced lymphopenia (RIL) (7). With growing interest in this topic driven by the emergence of immunotherapy, in recent years, several studies demonstrated an independent association between lymphopenia and detrimental survival in NSCLC (8–12). In addition, two recent studies in NSCLC patients observed that severe lymphopenia before the initiation of immunotherapy was associated with worse progression-free and overall survival outcomes (13, 14).

The apparent impact of lymphopenia on the efficacy of consolidative immunotherapy and survival provides a strong incentive to identify patients at high risk of severe lymphopenia who could potentially benefit from lymphopenia-mitigating strategies. Thus, before starting CRT, accurate prediction of the individual risk of developing severe lymphopenia during CRT would be of interest. One such elaborate model predicting grade ≥3 lymphopenia originated from The Christie (Manchester, UK) and was developed in 901 lung cancer patients, of whom 227 patients received concurrent CRT for NSCLC (11). External validation of the Christie model in 305 patients with esophageal cancer yielded a satisfactory *c*-statistic of 0.78 (11). A more simple prediction model (i.e., not requiring any dosimetric parameters) predicting grade 4 lymphopenia originated from MD Anderson Cancer Center (MDACC, Houston, Texas, USA) and was developed in 860 patients with esophageal cancer (15). External validation of the MDACC model in 219 patients with esophageal cancer in another country yielded a satisfactory *c*-statistic of 0.80 (16).

The Christie experience (11) suggested that a prediction model for lymphopenia developed in lung cancer could be used interchangeably in esophageal cancer. Although validated in other esophageal cancer cohorts, the MDACC model (15) has not yet been validated in patients with lung cancer. Therefore, the aim of this study was to externally validate and compare both the Christie and MDACC model for predicting grade ≥3 and grade 4 RIL during concurrent CRT in patients with stage III NSCLC.

## Methods

This single-center retrospective cohort study was approved by the institutional review board and the need for informed consent was waived. All patients had an institutional opt-out option upon first consultation in case they wished their data would not be used for research purposes. Reporting of this study was performed in accordance with the “Transparent Reporting of a Multivariable Prediction Model for Individual Prognosis or Diagnosis” (TRIPOD) guidelines (17).

### Study population

Consecutive patients who underwent concurrent CRT for stage III NSCLC at our comprehensive cancer center between February 2019 and November 2021 were eligible for inclusion. Patients who opted out for consenting to use their data for research were excluded. Some patients were treated at a satellite location of our hospital where no routine determination of absolute lymphocyte counts (ALCs) was performed and these patients were therefore excluded. In addition, patients were excluded in case a baseline ALC or ALC beyond the first 3 weeks of CCRT was lacking or in case therapy was discontinued in the first 2 weeks for issues unrelated to lymphopenia. Finally, a patient with an active hematologic malignancy (and associated high baseline ALC) was excluded. A detailed comparison of the inclusion and exclusion criteria of the development cohorts (11, 15) and the current validation cohort is provided in **Supplementary Table 1**.

### Treatment

Chemotherapy consisted of cisplatin 6 mg/m^2^ in 24 administrations (five times per week) and was administered as a bolus injection, 1–2 h before radiotherapy (18). All patients were treated with hypofractionated radiotherapy (24 × 2.75 Gy) up to 70 Gy (EQD2_10_) to the primary tumor and up to 60 Gy (EQD2_10_; 24 × 2.42 Gy) to the involved lymph nodes. Photon-based intensity-modulated radiotherapy was used in all patients and the two dose levels were achieved using a simultaneous integrated boost technique (19). A 4D-CT scan with intravenous contrast was acquired, from which a 3D-midposition-CT scan (MidP) was reconstructed. An ^18^F-FDG PET-CT scan was registered to the MidP to guide gross tumor volume (GTV) delineation of the primary tumor and pathologic lymph nodes in all patients. No CTV concept was applied. Subsequently, the GTVs were expanded to a planning target volume (PTV) using individualized margins according to the peak-to-peak respiratory amplitude movement of the primary tumor and lymph nodes.

### Predictors

The refined dosimetry-based Christie model predictors for grade ≥3 lymphopenia included higher age, lower baseline ALC, longer duration of radiotherapy, higher mean heart and lung doses, and higher thoracic vertebrae V20Gy (i.e., volume of thoracic vertebrae receiving ≥20 Gy) (11). In the more simple PTV-based MDACC model, predictors for grade 4 lymphopenia consisted of higher age, lower baseline ALC, and higher PTV in interaction with a lower body mass index (BMI) (15). For the current study, all of these predictors were collected from our institutional database in addition to other baseline characteristics (i.e., gender, year of treatment start, histology, tumor location, and clinical stage).

The original Christie prediction model (11) is defined by the following logistic regression formula, where *p* describes the individual risk to develop grade ≥3 lymphopenia:


$$\begin{array}{c} \log\left(\frac{p}{1-p}\right)=-4.654+0.019*Age-0.544*Baseline\_ALC\\ +0.435*chemotherapy[0=\text{no};1=\text{yes}]\\ +0.090*Radiotherapy\_duration\\ +0.028*Mean\_heart\_dose\\ +0.046*Mean\_lung\_dose\\ +0.014*Vertebrae\_V20Gy. \end{array}$$

The original MDACC prediction model (15) for the prediction of grade 4 lymphopenia is defined as follows:


$$\begin{array}{c} \log\left(\frac{p}{1-p}\right)=-22.845+0.021*Age-1.019*Baseline\_ALC\\ +0.516*BMI+3.579*\log\left(PTV\right)\\ -0.086*BMI*\log\left(PTV\right)+0.949*\;Photons\\ {}[0=\text{no},\text{ Protons};1=\text{yes},\text{ Photons}]. \end{array}$$

### Outcomes

ALC was routinely measured mostly twice per week (but at least once weekly) during concurrent CRT as part of routine blood examinations to evaluate hematologic and renal chemotherapy toxicity. The frequency of blood examinations in The Christie and MDACC cohorts was generally less and details are provided in **Supplementary Table 1**. The lowest measured ALC during CRT on a per-patient basis was defined as the nadir. The primary outcomes of grade ≥3 and grade 4 lymphopenia were defined as ALC nadirs during CRT of <0.5 and <0.2 K/µL, respectively, in accordance with the Common Terminology Criteria for Adverse Events (version 5). The studied outcome measure consisted of the external model performance in terms of discrimination and calibration of the Christie and MDACC models to predict the risk of grade ≥3 and grade 4 lymphopenia.

### Statistical analysis

A table with baseline characteristics was constructed. Univariable logistic regression analyses were performed to explore the crude associations of (baseline) variables with grade ≥3 and grade 4 lymphopenia. Next, for each patient, the individual predicted probabilities of grade ≥3 and grade 4 lymphopenia according to the Christie and MDACC prediction models were calculated. The discriminatory model performances were assessed by calculating external *c*-statistics and by plotting ROC curves. External model calibration performances (i.e., the agreements between predicted and observed proportions of grade ≥3 or grade 4 lymphopenia) were visually assessed in calibration plots using 3 equally sized risk groups (i.e., tertiles of predicted risks).

In case of miscalibration due to a different *a priori* risk (i.e., incidences) of grade ≥3 or grade 4 lymphopenia in the current cohort compared to the original Christie and MDACC development cohorts, the intercept was updated for each model and each outcome. This intercept was updated in such way that the sum of predicted probabilities was equal to the observed number of events (20). Model coefficients were not updated. Analyses were performed using SPSS version 27.0 (IBM Corp., Armonk, NY) and R version 3.5.1 (“rms” package). A *p*-value<0.05 was considered statistically significant.

## Results

From a total of 148 identified patients who underwent concurrent CRT for stage III NSCLC in the study period, 100 patients were eligible for analysis. The 48 patients were excluded because of the opt-out procedure (*n* = 3), concurrent CRT was administered at the satellite location of our hospital with no routine ALC values available (*n* = 16), baseline ALC values were missing (*n* = 24), ALC values beyond 3 weeks after start of treatment were missing (*n* = 2), therapy was discontinued early due to a severe COVID-19 infection or unexpected sudden death (*n* = 2), or an active hematologic malignancy was present (*n* = 1).

The majority of the 100 included patients were male (55%) and were diagnosed with a clinical T4 (50%) and/or N2–3 (84%) lung cancer. The predominant histologic tumor types were adenocarcinoma (48%) and squamous cell carcinoma (35%), and the primary tumor was mostly located in an upper lobe (67%). Baseline patient-, tumor-, and treatment-related characteristics are presented in **Table 1**.

**Table 1:** Baseline characteristics of 100 included patients.

	*n* (%)
Age (years)*	66.1 ± 8.4
**Male gender**	55 (55%)
BMI (kg/m^2^)*	25.3 ± 4.5
Year of treatment start	
2019	30 (30%)
2020	38 (38%)
2021	32 (32%)
Histology	
Adenocarcinoma	48 (48%)
Squamous cell carcinoma	35 (35%)
Other (large cell)	17 (17%)
Primary tumor lateralization	
Left sided	40 (40%)
Right sided	59 (59%)
Missing	1 (1%)
Primary tumor location	
Upper lobe	67 (67%)
Middle lobe	4 (4%)
Lower lobe	25 (25%)
Trachea/main bronchus	3 (3%)
*Missing*	1 (1%)
Clinical T-stage	
cT1	20 (20%)
cT2	18 (18%)
cT3	12 (12%)
cT4	50 (50%)
Clinical N-stage	
cN0	11 (11%)
cN1	5 (5%)
cN2	60 (60%)
cN3	24 (24%)
Overall clinical stage	
IIIA	46 (46%)
IIIB	39 (39%)
IIIC	15 (15%)
**Baseline ALC (K/µL)^†^**	1.71 [1.18–2.19]
**PTV (mL)^†^**	0.337 [0.204–0.503]
**Mean heart dose (Gy)^†^**	4.34 [1.93–8.62]
**Mean lung dose (Gy)^†^**	11.1 [9.16–13.2]
**Thoracic vertebrae V20Gy (%)^†^**	23.5 [14.8–32.4]

*Expressed as mean ± SD. ^†^Expressed as median [IQR]. ALC, Absolute lymphocyte count; BMI, Body mass index; PTV, Planning target volume.

**Figure 1:**
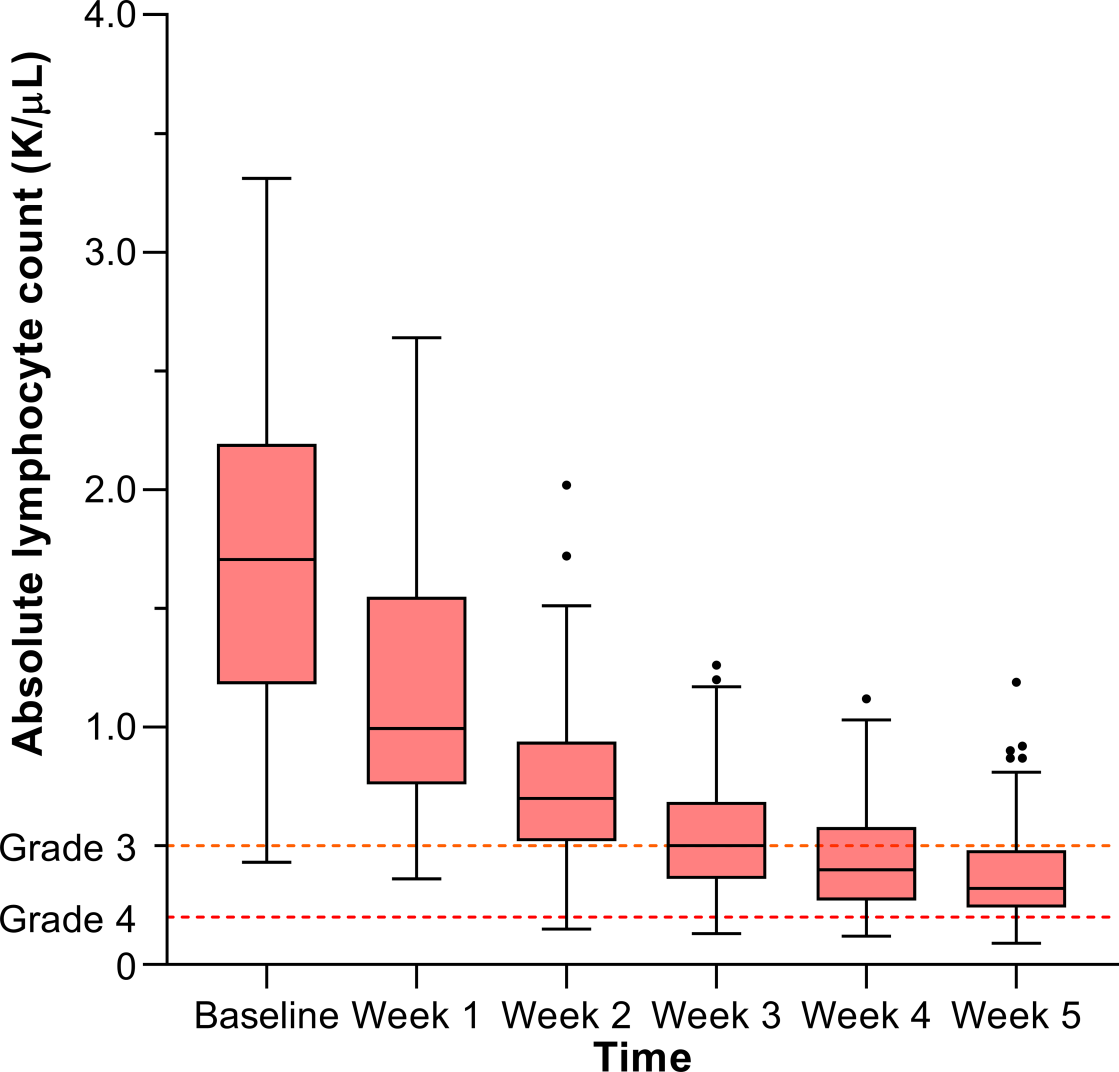
Lymphocyte counts over the course of treatment.

The course of ALC values over the time of treatment showed an overall declining trend and is illustrated in **Figure 1**. The median duration of treatment was 32 days [IQR: 31–33]. The ALC nadir was observed at a median of 30 days [IQR: 25–31] after the start of concurrent CRT, generally corresponding to the fifth week of treatment. Grade ≥3 lymphopenia during concurrent CRT occurred in 78 patients (78%), and among those patients, grade 4 lymphopenia was observed in 17 (17% of total).

Explorative logistic regression analysis for grade ≥3 and grade 4 lymphopenia is presented in **Table 2**. Significant univariable associations were observed for baseline ALC and PTV with both grade ≥3 and grade 4 lymphopenia. For the 78 patients with versus 22 patients without grade ≥3 lymphopenia, median baseline ALC was 1.53 [IQR: 1.13–1.93] versus 2.36 [IQR: 1.93–2.62] K/µL, respectively, and median PTV was 392 [IQR: 237–522] versus 203 [132–347] mL, respectively. For the 17 patients with versus 83 patients without grade 4 lymphopenia, median baseline ALC was 1.37 [IQR: 1.02–1.73] versus 1.83 [IQR: 1.24–2.33] K/µL, respectively, and median PTV was 499 [IQR: 325–718] versus 296 [IQR: 190–496] mL, respectively. In addition, right-sided tumor lateralization and mean lung dose appeared significantly associated with grade ≥3 lymphopenia.

**Table 2:** Exploratory univariable logistic regression analyses for grade ≥3 and grade 4 lymphopenia.

	Grade ≥3 lymphopenia		Grade 4 lymphopenia	
	OR (95% CI)	*p*-value	OR (95% CI)	*p*-value
**Age (years)**	1.04 (0.98–1.10)	0.170	1.00 (0.94–1.07)	0.988
**Male gender**	1.64 (0.63–4.24)	0.311	2.23 (0.72–6.90)	0.163
**BMI (kg/m^2^)**	1.01 (0.91–1.12)	0.910	0.95 (0.84–1.07)	0.388
Histology				
Adenocarcinoma	Ref		Ref	
Squamous cell carcinoma	1.44 (0.48–4.35)	0.521	1.74 (0.56–5.34)	0.337
Other (large cell)	0.71 (0.21–2.47)	0.594	0.78 (0.15–4.19)	0.773
**Right-sided tumor lateralization**	4.46 (1.61–12.3)	0.004*	0.96 (0.33–2.78)	0.943
Primary tumor location				
Upper lobe	Ref		Ref	
Other	2.66 (0.82–8.64)	0.103	1.13 (0.38–3.38)	0.825
Clinical T-stage				
cT1–2	Ref		Ref	
cT3–4	1.49 (0.57–3.88)	0.416	1.15 (0.39–3.42)	0.801
Clinical N-stage				
cN0–1	Ref		Ref	
cN2–3	2.55 (0.81–8.05)	0.110	0.87 (0.22–3.45)	0.839
Overall clinical stage				
IIIA	Ref		Ref	
IIIB-C	3.25 (1.19–8.88)	0.022*	1.27 (0.44–3.65)	0.662
**Baseline ALC (K/µL)**	0.24 (0.11–0.53)	0.001*	0.25 (0.09–0.69)	0.007*
**Log(PTV) [mL]**	4.53 (1.83–11.3)	0.001*	5.86 (1.83–18.7)	0.003*
**Mean heart dose (Gy)**	1.08 (0.96–1.20)	0.195	1.01 (0.92–1.11)	0.853
**Mean lung dose (Gy)**	1.22 (1.05–1.40)	0.008*	1.10 (0.94–1.28)	0.226
**Thoracic vertebrae V20Gy (%)**	1.04 (0.99–1.08)	0.088	1.02 (0.98–1.06)	0.381
**Radiotherapy duration (days)**	0.86 (0.63–1.16)	0.857	0.94 (0.66–1.33)	0.713

*Statistically significant univariable association with the outcome. ALC, Absolute lymphocyte count; BMI, Body mass index; CI, Confidence interval; OR, Odds ratio; PTV, Planning target volume.

**Figure 2:**
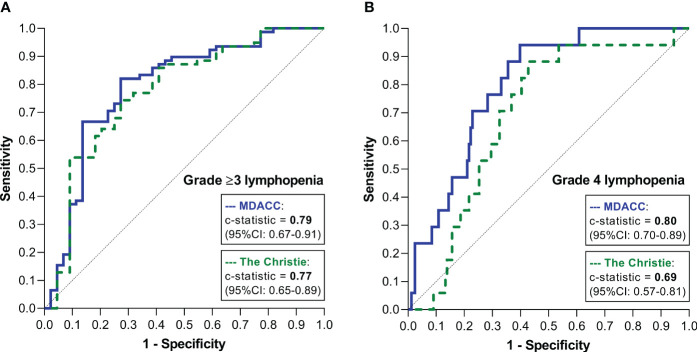
ROC curves demonstrating the discriminatory performances of the two models for predicting grade ≥3 lymphopenia **(A)** and grade 4 lymphopenia **(B)**.

For prediction of grade ≥3 lymphopenia, application of the Christie and MDACC models yielded *c*-statistics of 0.77 (95% CI: 0.65–0.89) and 0.79 (95% CI: 0.67–0.91), respectively (**Figure 2A**). For prediction of grade 4 lymphopenia, the Christie and MDACC models yielded *c*-statistics of 0.69 (95% CI: 0.57–0.81) and 0.80 (95% CI: 0.70–0.89), respectively (**Figure 2B**).

Application of the Christie model for predicting grade ≥3 lymphopenia resulted in an uncorrected calibration in which the predicted risk consistently underestimated the observed risk. Therefore, the model was updated by intercept correction to take into account the higher *a priori* risk of grade ≥3 lymphopenia in the current cohort compared with the development cohort (i.e., 78% versus 55% (11); **Supplementary Figures 1A, B**). In contrast, the MDACC prediction model for predicting grade 4 lymphopenia resulted in an uncorrected calibration in which the predicted risk consistently overestimated the observed risk. Therefore, the model was updated by intercept correction to take into account the lower *a priori* risk of grade 4 lymphopenia in the current cohort compared with the development cohort (i.e., 17% versus 37% (15); **Supplementary Figures 1C, D**). Separately, the intercept of the Christie model (developed for predicting 55% grade ≥3 lymphopenia (11)) was also adjusted to the currently observed incidence of grade 4 lymphopenia (17%), and the intercept of the MDACC model (developed for predicting 37% grade 4 lymphopenia (15)) was similarly adjusted to the observed incidence of grade ≥3 lymphopenia.

**Figure 3:**
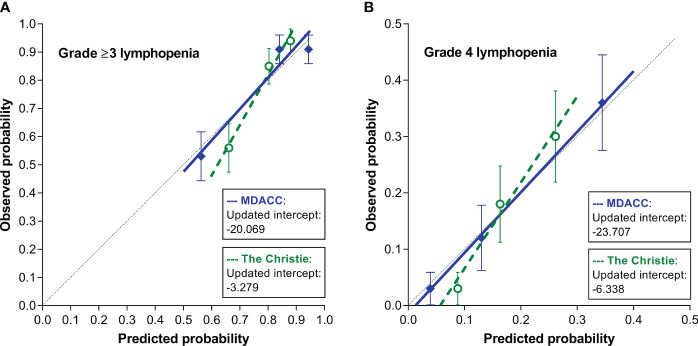
Calibration plots demonstrating the agreement between the predicted risks by the 2 models and the observed risks of grade ≥3 lymphopenia **(A)** and grade 4 lymphopenia **(B)**.

The resulting agreement between predicted and observed risks (i.e., calibration performance) of the models was visually assessed (**Figure 3**). For predicting grade ≥3 lymphopenia, the Christie and MDACC models demonstrated moderate and good agreement, respectively (**Figure 3A**). For predicting grade 4 lymphopenia, the Christie and MDACC models demonstrated moderate and excellent agreement, respectively (**Figure 3B**).

## Discussion

This study demonstrated that the majority (78%) of patients with stage III NSCLC experience grade ≥3 lymphopenia during concurrent CRT, and in 17% of patients, the ALC drops as low as<0.2 K/mL (i.e., grade 4). The performance of the simple PTV-based MDACC prediction model for severe RIL (15) in our current external cohort appeared superior in terms of discrimination and calibration in comparison to the more refined dosimetry-based Christie model (11). In fact, the MDACC prediction model developed in esophageal cancer for grade 4 RIL (15) demonstrated good external performance in our setting of concurrent CRT for patients with stage III NSCLC with a *c*-statistic of 0.80 and excellent calibration. In addition, after intercept adjustment to the *a priori* risk, the MDACC model appeared capable of satisfactorily distinguishing patients who will experience grade ≥3 lymphopenia versus those who will not with a *c*-statistic of 0.79 and good calibration. The herein reported external model performance (in another center in another country) suggests good overall generalizability of the model.

As the MDACC lymphopenia model developed and validated in esophageal cancer appeared compatible in lung cancer, the model could potentially serve as a generalized thoracic cancer risk tool for predicting severe lymphopenia. Similarly, the Christie lymphopenia model developed in lung cancer was previously validated in a cohort of esophageal cancer as well with satisfactory results (11). As such, these current and previous findings can help encourage increased collaboration of investigators and clinicians in the fields of esophageal cancer and lung cancer to jointly elucidate the impact of lymphopenia, improve risk prediction, and study strategies to mitigate the lymphopenia risk.

Because the simple PTV-based MDACC model does not contain dosimetric variables, the model cannot aid in optimizing radiotherapy planning parameters for lymphopenia mitigation. For the important goal of lymphopenia mitigation by adjusting radiotherapy plans, refined dosimetry-based prediction models such as the Christie model are required. However, this study demonstrates that applying such a complex dosimetry-based model in an external setting carries more risk of suboptimal performance (i.e., appears less robust across varying disease sites, lymphopenia outcome definitions, and care settings). Therefore, for moving forward with radiotherapy planning optimization aimed at reducing the risk of severe lymphopenia, further improvement and external validation of dosimetry-based models is desired.

The initial poor calibration of the prediction models is explained by the lower incidence of grade 4 lymphopenia in the current lung cancer cohort (13%) in comparison with the esophageal cancer cohorts from MDACC used for development (37%) (15), and our higher incidence of grade ≥3 lymphopenia (78%) in comparison to the Christie development cohort (55%) (11). After intercept correction to adjust for this varying incidence, the calibration of the MDACC model in our external lung cancer cohort was good to excellent, revealing that the same predictors in esophageal cancer hold their predictive value in lung cancer.

The relatively high incidence of grade 4 lymphopenia in esophageal cancer has been confirmed in multiple series (16, 21–23), and is attributed to the major collateral irradiation of large pools of lymphocytes (e.g., heart, lungs, and aorta) in typical esophageal cancer radiotherapy fields (7, 22). Besides the major role of blood pool irradiation, the risk and depth of RIL may also be affected (to a lesser extent) by irradiation of the spinal column that contains a significant portion of the hematopoietic potential in adults and replenishes the circulating lymphocyte pool (7, 22). The statistical power of lymphopenia risk modeling in esophageal cancer is strengthened by the high number of events, but limited by the relatively small variation in tumor location and thus in dosimetric parameters. In contrast, risk modeling in lung cancer is hampered by the lower incidence of grade 4 lymphopenia, but strengthened by the larger variation in dosimetric parameters by greater tumor location variability. Therefore, combining knowledge and data from both esophageal and lung cancer populations carries the potential to overcome current limitations.

Although the typical depletion of lymphocytes over the course of treatment is typical for radiotherapy-only as well as CRT cohorts, the addition of chemotherapy does result in an even lower ALC nadir (24). A study comparing different doublet platinum-based chemotherapy regimens (i.e., cisplatin-etoposide, cisplatin-docetaxel, carboplatin-paclitaxel, and carboplatin-docetaxel) found an equal 88%–89% grade ≥3 lymphopenia rate for each regimen (25). Since reported incidence rates of grade ≥3 lymphopenia in stage III NSCLC patients undergoing 3-weekly doublet platinum-based chemotherapy vary from 49% to 89% (7, 9–11, 22, 25), the incidence in the current study of 78% does not appear different with our daily low-dose cisplatin regimen.

Before commencing treatment, the MDACC prediction model allows the identification of individual patients at high risk for severe lymphopenia. These patients may benefit from lymphopenia-mitigating strategies. Examples of such strategies include further hypofractionation, sparing lymphocyte-rich organs in radiation treatment planning, or reducing PTV by minimizing radiotherapy margins through modern daily online adaptive radiotherapy (e.g., using MR-linac) (7, 11, 26). In addition, a significant lymphocyte-sparing effect of proton beam therapy, through decreasing the integral body dose, has been convincingly demonstrated in esophageal cancer and more recently also in lung cancer (7, 11, 12, 26–28). In the recent systematic review-based LymphoTEC initiative, dose constraints were described that can be used in clinical practice and future studies to limit the risk of RIL and possibly improve oncologic outcomes (29). Experimental attempts to attenuate lymphopenia included isolating lymphocytes before treatment with reinfusion upon treatment completion (which appeared feasible and safe, but not effective) (30), or administering interleukins (e.g., IL-2, IL-7, and IL-15) essential for lymphocyte proliferation and survival with promising results in pilot studies (31).

Besides inherent shortcomings resulting from the retrospective design of the current study, some other limitations require mention. First, no causal inferences can be made between predictors and the outcome of severe lymphopenia since this was no intervention study. The observational design allows for concluding strong associations only. Second, a larger sample size could have increased the precision of estimations and may have allowed for further model improvements. Third, in the analyses on the performance of the Christie model for predicting grade 4 lymphopenia and the MDACC for grade ≥3 lymphopenia, the studied outcome was (intentionally) defined differently from how it was defined in the original publications. Intercept corrections were applied to adjust for the large differences in *a priori* risks between grade ≥3 and grade 4 lymphopenia, but model coefficients were kept the same. This approach assumed that the relative contribution of predictors would be similar for grade ≥3 and grade 4 lymphopenia, but this assumption might not completely hold. However, this method was chosen because further model updates (e.g., adjusting model coefficients) would imply developing a new prediction model, which, in turn, would require another internal and external validation. Fourth, the survival impact of lymphopenia was not studied here as follow-up was too short for this recent cohort. This study is strengthened by the homogeneous study cohort and the frequent (i.e., twice-weekly) determination of ALC values with only very few missing values.

In conclusion, 78% and 17% of patients with stage III NSCLC who undergo concurrent CRT develop grade ≥3 and grade 4 lymphopenia, respectively. The simple PTV-based MDACC prediction model (15) for grade 4 lymphopenia developed in patients with esophageal cancer demonstrated good external performance in the setting of lung cancer, and outperformed the more refined dosimetry-based Christie prediction model (11). Good to excellent discriminative ability and agreement between predicted and observed risk were observed. Before treatment, the MDACC model can identify thoracic cancer patients at high risk of severe lymphopenia who might benefit most from lymphopenia-mitigating strategies, which may ultimately improve survival. To optimize radiotherapy planning with the purpose of reducing the risk of severe lymphopenia, further improvement and external validation of dosimetry-based models (such as the Christie model) is desired.

## Data availability statement

The raw data supporting the conclusions of this article will be made available upon request, without undue reservation.

## Ethics statement

The studies involving humans were approved by Netherlands Cancer Institute Institutional Review Board. The studies were conducted in accordance with the local legislation and institutional requirements. Retrospective data collection. Nothing was asked from the patients. Data was handled anonymously.

## Author contributions

PR: Conceptualization, Data curation, Formal Analysis, Investigation, Methodology, Project administration, Validation, Visualization, Writing – original draft. CJ-C: Data curation, Investigation, Methodology, Writing – review & editing. BS: Data curation, Investigation, Methodology, Writing – review & editing. MR: Data curation, Investigation, Writing – review & editing. SL: Investigation, Methodology, Validation, Writing – review & editing. AA: Investigation, Methodology, Validation, Writing – review & editing. JB: Data curation, Formal Analysis, Investigation, Methodology, Supervision, Writing – review & editing. J-JS: Conceptualization, Formal Analysis, Investigation, Methodology, Supervision, Validation, Writing – review & editing.

## Supplementary material

SUPPLEMENTARY FIGURE 1 Calibration plots of the Christie model for predicting grade ≥3 lymphopenia before **(A)** and after **(B)** correcting the model intercept to match the a-priori risk of 78%. Calibration plots of the MDACC model for predicting grade 4 lymphopenia before **(C)** and after **(D)** correcting the model intercept to match the a-priori risk of 17%.
