# Supplementary material for: Severe radiation-induced lymphopenia during concurrent chemoradiotherapy for stage III non-small cell lung cancer: external validation of two prediction models
Source: Front Oncol. 2023 Nov 9;13:1278723. doi: 10.3389/fonc.2023.1278723 (PMC10665840; doi:10.3389/fonc.2023.1278723)
Supplement: Supplementary file 2 [file Table_1.docx]

| **Supplementary Table 1** Description of study populations of the development and validation cohorts. | | |
| --- | --- | --- |
| **The Christie development cohort** | **MDACC development cohort** | **Current validation cohort** |
| *Inclusion criteria:*  - Stage I-III NSCLC and SCLC patients  - Treated with definitive RT (with or without chemotherapy)  - From 2005 to 2017 | *Inclusion criteria:*  - Stage I-III esophageal cancer patients  - Who underwent CCRT  - From 2004 to 2017 | *Inclusion criteria:*  - Stage III NSCLC patients  - Who underwent CCRT  - From 2019 to 2021 |
| *Exclusion criteria:*  - No baseline ALC available  - Baseline ALC <0.5 K/µL  - Not a single ALC during RT available | *Exclusion criteria:*  - Not any ALC data available  - No baseline ALC available  - Less than 3 ALCs during CCRT available  - Radiation dose other than 50.4 Gy  - Simultaneous second primary tumor  - Hematologic malignancy | *Exclusion criteria:*  - Not any ALC data available  - No baseline ALC available  - No ALC beyond 3 weeks available  - Discontinuation of CCRT in first 2 weeks  - Hematologic malignancy |
| AC: Adenocarcinoma. ALC: Absolute lymphocyte count. CCRT: Concurrent chemoradiotherapy. MDACC: MD Anderson Cancer Center. NSCLC: Non-small cell lung cancer. RT: Radiotherapy. SCC: Squamous cell carcinoma. SCLC: Small cell carcinoma. | | |
